# The ChatGPT (Generative Artificial Intelligence) Revolution Has Made Artificial Intelligence Approachable for Medical Professionals

**DOI:** 10.2196/48392

**Published:** 2023-06-22

**Authors:** Bertalan Mesko

**Affiliations:** 1 The Medical Futurist Institute Budapest Hungary; 2 Department of Behavioural Sciences Semmelweis University Budapest Hungary

**Keywords:** artificial intelligence, digital health, future, technology, ChatGPT, medical practice, large language model, language model, generative, conversational agent, conversation agents, chatbot, generated text, computer generated, medical education, continuing education, professional development, curriculum, curricula

## Abstract

In November 2022, OpenAI publicly launched its large language model (LLM), ChatGPT, and reached the milestone of having over 100 million users in only 2 months. LLMs have been shown to be useful in a myriad of health care–related tasks and processes. In this paper, I argue that attention to, public access to, and debate about LLMs have initiated a wave of products and services using generative artificial intelligence (AI), which had previously found it hard to attract physicians. This paper describes what AI tools have become available since the beginning of the ChatGPT revolution and contemplates how it they might change physicians’ perceptions about this breakthrough technology.

## The ChatGPT Revolution

In November 2022, OpenAI publicly launched its large language model (LLM), ChatGPT [1]. It reached the milestone of having over 100 million users in only 2 months. In comparison, reaching the same milestone took TikTok and Instagram 9 months and more than 2 years, respectively. In March 2023, OpenAI had already released a new iteration called GPT-4 that was claimed to be 100 times better than the previous version.

LLMs have seen rapid advancements and practical applications in various industries from marketing and information technology to publishing and even health care [2].

These models can generate human-like text, assist in diagnosing conditions based on medical records, and even suggest treatment options or plans. Given the potential implications for patient outcomes and public health, as well as the impact on the jobs of medical professionals, LLMs have attracted much attention.

LLMs have been shown to be used in a myriad health care–related tasks and processes. Examples include education and research [3], analyzing electronic health records [4], oncology [5], cardiology [6], writing discharge summaries [7], and medical journalism [8]. The number of use cases has skyrocketed, as well as the challenges and issues around the use of this particular technology. The ethics of its use have been analyzed in depth [9]: LLMs have been hallucinating references; their role as a potential coauthor of research papers has been debated; it has been shown that such models can pose a cybersecurity threat [10]; and they can be biased [11].

Nevertheless, in this paper, I argue that attention to, public access to, and debate about LLMs have initiated a wave of products and services using generative artificial intelligence (AI), which had previously found it hard to attract physicians.

There are events of a certain magnitude that can lead to significant changes in a field, especially regarding the attitude of people experiencing them. The COVID-19 pandemic has led to an unprecedented level of patients and physicians adopting technologies such as remote care and at-home laboratory testing [12]. Advances in cloud computing led to new discoveries and developments in AI in the 2010s. The release of LLMs can mark a similarly impactful event that leads to physicians gaining first-hand experience with AI-based tools and technologies.

No matter how many studies, clinical trials, regulatory approvals, and product announcements keep on becoming available about AI’s role in medicine and health care, adoption rates have been lagging behind [13,14]. One of the reasons for this has been limited access to AI technologies. There have been hundreds of AI applications with regulatory approval; however, they were only available in limited health care institutions to a select group of physicians [15].

This paper describes what AI tools have become available since the beginning of the ChatGPT revolution and contemplates how they might change physicians’ perceptions of this breakthrough technology.

## The Swarm of Generative AI Tools

In principle, tasks in a physician’s job that are repetitive in their nature and are data-based are prone to being automated. Most of the AI-based medical technologies that have received regulatory approval are built on this idea and address one specific task, mostly in radiology, cardiology, and oncology.

Generative AI has brought this to a new level. Generative AI refers to a category of AI models that are capable of generating new data samples by learning the underlying patterns and structures of a given data set. These models, including generative adversarial networks (GANs) and generative pretrained transformer (GPT)-like architectures, have demonstrated prowess in a wide range of applications, including image synthesis, natural language processing, and drug discovery. As generative AI continues to advance, it holds the potential to revolutionize industries by enabling the creation of novel and customizable content, accelerating research, and expanding the scope of human creativity.

Since the end of 2022, basic AI tools and services using generative AI have become widely accessible. This might mark the beginning of AI becoming used in practice for the average medical professional.

Even health care companies have started to integrate LLMs into their services. Examples include Microsoft’s Nuance bringing ChatGPT into its medical note-taking tool; Nabla (Nabla SRL) using ChatGPT to have conversations with patients; and Epic introducing LLMs to their electronic health record software [16-18].

## Examples of AI Tools for Physicians

### Building a Website With AI

There are AI-based website builders that can create a brand-new website in a few minutes. Users can choose between design themes and a number of functionalities. Some of the companies even offer more advanced frameworks, such as web shops and responsive sites that automatically adjust to the device—mobile or computer—the visitor uses [19].

The output currently is not as versatile or customized as a site built by a team of professionals, and these services have limitations with search engine optimization, but a physician building a website for their practice in minutes is still an impressive feat.

### Creating Videos With AI

LLMs are capable of coming up with ideas for video content and even writing the scripts (eg, with prompts such as “write a 5-minute script about the importance of the flu shot for the elderly”). Revoicer (Revoicer Ltd), a text-to-speech algorithm, can transform the script into audio content, and tools like Yepic (Yepic AI Ltd) and Synthesia (Synthesia Ltd) can generate a video featuring a synthetic human. While synthetic voices and humans do not reflect the original, these tools still allow users to create informative video content without having a content production team at hand for a fraction of the cost of hiring all these professionals [20].

### Designing Presentations With AI

Creating presentations for meetings and conferences can be cumbersome even if clinicians are experienced with presentation tools like PowerPoint (Microsoft Corp) or Prezi (Prezi Inc). There are AI presentation tools that design stylish presentations from primary inputs. Physicians can choose a template and input basic data, information, and facts, and AI will do the rest by formatting the slides and offering visuals, animations, or voice-overs. The results can be somewhat generic—but in most cases are still more attractive than what most physicians could make by themselves [21].

### AI as a Medical Scribe

A growing number of algorithms claim to record and transcribe meetings or conversations automatically, analyze the content, and provide an easy-to-understand, searchable report. Some of these solutions are specifically designed for medical use, while others target a more general audience. Companies targeting health care users offer complex services, such as AI transcribing a consultation that is then reviewed by a human professional for accuracy. Clear limitations are that general transcription tools might pose a risk when used in medical settings, while those specifically designed for medical uses tend to be on the expensive side. However, using such tools might relieve burdens on medical professionals and reduce excessive administrative tasks, which are a major cause of physician burnout [22].

### Creating Social Media Posts, Frequently Asked Questions, and Other Informational Content for Patients With AI

Generative AI, especially LLMs and AI image generators such as DALL-E (OpenAI) and MidJourney (MidJourney Inc), can be used to provide written and image-based content for patients. ChatGPT can assist physicians in crafting social media posts, general informational materials, and brochures aimed at educating patients. While physicians need to verify every detail these generated posts contain, using AI can still save considerable time in the process.

### Designing Logos and Images

AI image generators like DALL-E and MidJourney can generate images based on text prompts. Such images can be used as logos for medical practices, visualizations for social media posts, and to help deliver messages on websites.

### Medical Research and Literature Analysis

Tools like Semantic Scholar (Allen Institute for AI) use AI to help physicians stay up-to-date with the latest research findings by analyzing and summarizing relevant articles, making it easier to keep abreast of medical advancements.

The difference between these and the previously regulated AI-based medical technologies is that the tools described above are widely accessible, do not require any prior technical training, and are not dedicated to niche areas such as specific medical specialties.

## The Impact of Generative AI on the Accessibility of AI for Medical Professionals

Using AI was previously possible in research groups dedicated to this technology, in health care settings where certain tools were available, and for selected individuals who were fortunate enough to receive access to them. The widespread release of LLMs and other AI tools using generative AI has increased accessibility for medical professionals to test this breakthrough technology. It has also come with additional benefits.

### Democratization of AI Technology

Open-source projects and accessible platforms have allowed developers and researchers to contribute to AI advancements, fostering a community-driven approach that has enhanced the growth and reach of AI tools in the medical field. AutoGPT represents one of the most popular directions [23].

### Cost-Effectiveness

The affordability of AI solutions has enabled medical professionals to adopt these tools in their practice without incurring prohibitive costs. This has been essential in bridging the gap between cutting-edge research and its practical application in health care settings.

### Improved Data Processing and Analysis

LLMs excel at handling vast amounts of unstructured data, such as clinical notes and medical literature, which has facilitated the extraction of valuable insights and improved decision-making processes for medical professionals.

### Enhanced Communication and Collaboration

AI-powered tools can streamline communication between health care providers, patients, and interdisciplinary teams, promoting a more collaborative environment and fostering better patient outcomes.

### Continuous Learning and Adaptation

LLMs have the capacity to learn from new data and adapt to the evolving needs of medical professionals, thus offering more effective and targeted solutions in real time. Physicians can ask ChatGPT to provide summaries, explanations, and key points from recent medical literature and can present hypothetical patient cases or real anonymized cases and seek guidance on differential diagnoses, treatment options, and potential risks, further enhancing their clinical decision-making abilities. Physicians can even request ChatGPT to generate quiz questions or self-assessment exercises on specific medical topics to test their knowledge and identify areas for improvement.

## Generative AI Raises More Questions Than We Can Answer

While it is simple to see the benefits of a generation of medical professionals obtaining real-life experience with a technology like AI, the journey ahead raises more questions than we can answer now. Here, I summarize those questions with the highest importance.

There are strict regulations on the way AI technologies designed for a medical or health care–related purpose can access patient and medical databases, but there are none for generative AI tools made for a general audience without any medical purpose. If physicians use such tools, they are left alone with no guidance about how to deal with patient privacy or legal responsibilities.

Moreover, as LLMs tend to generate not only responses but also the resources they base their responses on, physicians need to verify every detail that comes from using generative AI.

Currently, no clinical guideline is available on their use and how to implement them in medical practice.

There is an ongoing debate about who owns copyright on AI outputs, such as text or images. If a physician uses an AI image generator to create a new logo for their website, in theory, anyone can use the same logo unless the user pays for the exclusive rights. The same issue stands for text made by generative AI for physicians to use in their research papers, as well as the presentations and videos created with these tools. There have been attempts from legal experts to provide solutions for this challenge, but there is no consensus yet [24].

As AI has finally become a tool for the masses, and it has become increasingly hard for physicians to ignore its use whether it is for medical, research or personal purposes, properly preparing medical professionals for its advantages and risks has become a timely challenge of crucial importance. Medical curricula, guidelines of medical associations, and the general discussion about AI’s future role in our profession must adjust accordingly.

Medical curricula could involve teaching prompt engineering to provide knowledge, skills, and a mindset for medical professionals to become proficient users of generative AI. Medical associations should provide a path forward for using such AI tools in the practice of medicine while keeping the values of evidence-based medicine and the importance of patient design in mind. And even more importantly, policymakers today face the challenge of not only designing policies and regulations for the generative AI tools medical professionals can use today, but also for the next versions and iterations that might involve analyzing other types of data, from image and video to sound and documents.

This is certainly an unprecedented challenge that requires relatively quick turnaround from all decision-makers in health care.

## Abbreviations

AI: artificial intelligence
GAN: including generative adversarial network
GPT: generative pretrained transformer
LLM: large language model
